# From Chip Size to Wafer-Scale Nanoporous Gold Reliable Fabrication Using Low Currents Electrochemical Etching

**DOI:** 10.3390/nano10112321

**Published:** 2020-11-23

**Authors:** Pericle Varasteanu, Cosmin Romanitan, Alexandru Bujor, Oana Tutunaru, Gabriel Craciun, Iuliana Mihalache, Antonio Radoi, Mihaela Kusko

**Affiliations:** 1 National Institute for Research and Development in Microtechnology (IMT-Bucharest), 126A Erou Iancu Nicolae Street, 077190 Voluntari, Romania; cosmin.romanitan@imt.ro (C.R.); alexandru.bujor@imt.ro (A.B.); oana.tutunaru@imt.ro (O.T.); gabriel.craciun@imt.ro (G.C.); iuliana.mihalache@imt.ro (I.M.); antonio.radoi@imt.ro (A.R.); 2 Faculty of Physics, University of Bucharest, 405 Atomistilor Street, 077125 Magurele, Romania; 3 Faculty of Chemistry, University of Bucharest, 90-92 Panduri Street, 050663 Bucharest, Romania

**Keywords:** nanoporous gold, large-scale fabrication, absorbance, X-ray diffraction, electrochemical impedance spectroscopy, scanning electrochemical microscopy

## Abstract

We report a simple, scalable route to wafer-size processing for fabrication of tunable nanoporous gold (NPG) by the anodization process at low constant current in a solution of hydrofluoric acid and dimethylformamide. Microstructural, optical, and electrochemical investigations were employed for a systematic analysis of the sample porosity evolution while increasing the anodization duration, namely the small angle X-ray scattering (SAXS) technique and electrochemical impedance spectroscopy (EIS). Whereas the SAXS analysis practically completes the scanning electronic microscopy (SEM) investigations and provides data about the impact of the etching time on the nanoporous gold layers in terms of fractal dimension and average pore surface area, the EIS analysis was used to estimate the electroactive area, the associated roughness factor, as well as the heterogeneous electron transfer rate constant. The bridge between the analyses is made by the scanning electrochemical microscopy (SECM) survey, which practically correlates the surface morphology with the electrochemical activity. The results were correlated to endorse the control over the gold film nanostructuration process deposited directly on the substrate that can be further subjected to different technological processes, retaining its properties. The results show that the anodization duration influences the surface area, which subsequently modifies the properties of NPG, thus enabling tuning the samples for specific applications, either optical or chemical.

## 1. Introduction

Nanoporous gold (NPG) has been known since the ancient times; the South American population and eastern part of Europe throughout the centuries used to etch (dealloy) and polish the surface of gold–copper alloys to create the illusion of bulk gold of shininess, a process known as depletion gilding or gold colouration (*mise-en-couleur*) [1]. The percolating structure of NPG, consisting of a matrix of interconnected gold ligaments and voids, was not known until 1960, when transmission electron microscopy was employed to study the corrosion of gold (Au) alloys [2].

Therefore, NPG preserves the properties of gold, such as high conductivity, good chemical stability, as well as biocompatibility, but also presents significant advantages, mainly determined by the large surface to volume ratio. Consequently, it becomes a good candidate for improving next generation chemical/biochemical sensors [3,4,5]. Thus, immunosensors, enzyme-based biosensors, or DNA sensors are only a few examples of sensors where an increase from 2 to 1000 times of the surface area in comparison with the planar gold counterpart of an equivalent geometric area would improve sensitivity and lower detection limits [6] Besides, Faradaic current scales linearly with the electrode area, which means that the NPG electrodes outperform the flat gold electrodes in terms of the amount of reagents immobilized on surface, higher signal to noise ratio, and smaller impedance [7]. In addition, NPG electrodes have recently been introduced as good current collectors in supercapacitors, firstly because they provide a large surface area to deposit active materials and guarantee effective charge transport [8], but also because they enhance the specific capacitance of poor-conductive active materials [7,9]. As for plasmonic sensing, NPG provides simple excitation schemes of strong plasmonic resonances localized on the nanoporous structure ligaments, with the resulting plasmon-induced electric field being the primary mechanism in surface-enhanced spectroscopy, including surface-enhanced Raman scattering (SERS), surface-enhanced infrared absorption (SEIRA), and surface-enhanced fluorescence (SEF) [10]. Furthermore, it was shown recently that NPG could be used as a high performance plasmonic biosensor in the near infrared spectral region (NIR), where the flat gold platforms exhibit low performances [10,11]. The catalytic activity is originated by the molecular oxygen activation, where the low coordinated gold atoms increase the strength of the oxygen binding [12]. Thus, high catalytic activity was reported towards CO oxidation of [13,14], but also hydrogen oxidation [15] or selective oxidation of alcohols [4]. An intriguing feature of NPG is the catalytic activity without additional supporting transition metal oxides nanoparticles [13]. Furthermore, taking into account that the upper limit of gold nanoparticles that could sustain catalytic activity is 5 nm [16], the ligament size larger than 10 nm seems to be too large for catalytic performances, but it is successfully compensated by the presence of steps, kinks, surface defects, twin boundaries, and dislocations in the NPG architecture [17,18,19].

Even though the gold nanostructures for various applications could be fabricated using lithographic techniques, the expensive equipment, processing cost, and small nanostructured area have slowed down the development of these structures. Moreover, it was shown that the NPG structures exhibit the same performances as the structures obtained from electron beam lithography [20], which means that the higher cost of fabrication and small area of the EBL fabricated gold nanostructures could be overcome by the NPG in several applications. The most used methods to fabricate NPG are dealloying [21] and templating [22]. In general, a binary gold alloys with different atomic weights are employed in dealloying, where, by chemical or electrochemical corrosion, the less noble metal is selectively removed from the compound, leading to a “sponge-like” structure with voids and ligaments [4,17,23]. Porosity depends on the atomic weight ratio in the alloy, thickness, and corrosion time. Templating represents deposition of the gold over an artificial or natural matrix with different sizes, with the porous structure being obtained after removing the template [24]. Several drawbacks are associated with these techniques, such as the increased stress within the structure, which might lead to cracks that can be detrimental especially for further technological processing or sensing applications [25], or the residual less noble metal after dealloying that artificially increases the double layer capacitance of the porous film [26,27]. Last, but not the least, the technology is problematic when the fragile, difficult to handle dealloyed leaves are then transferred onto a substrate (e.g., silicon wafers) that would be further subjected to the different photolithographic processes. Deposition of the alloy film directly by sputtering on the processable substrate is an alternative, but it significantly increases the fabrication cost. An attempt to obtain NPG directly from thin metallic gold films was realized using electrochemical porosification under high anodic bias up to 40 V [3,28]. However, a very high-bias regime induces local heating effects that focus charge carriers to the pore tips, considerably enlarging their diameters and imposing a limited process duration. Herein, based on our previous experience in fabricating porous silicon [29], a porosification procedure for Au was established using a constant applied current that can be generated with maximum 2.5 V bias, with the process being monitored after different periods of time, from 200 to 1000 s, when, lately, the entire gold film was deeply nanostructured. The obtained NPG films were characterized by means of scanning electron microscopy and correlated with the X-ray diffraction studies in order to obtain morpho-structural information (i.e., porosity, ligament diameter, and surface area) and to propose a mechanism of porosification under constant current anodization. Electrochemical investigations were further conducted to examine and visualize the active surface area in comparison with the flat gold. Not only does the process allow a good control over the NPG morpho-structural, optical, and electrochemical properties, it is also a fast, scalable, and cost-effective method, which is always desirable when talking about device manufacturing.

## 2. Materials and Methods 

**Figure 1:**
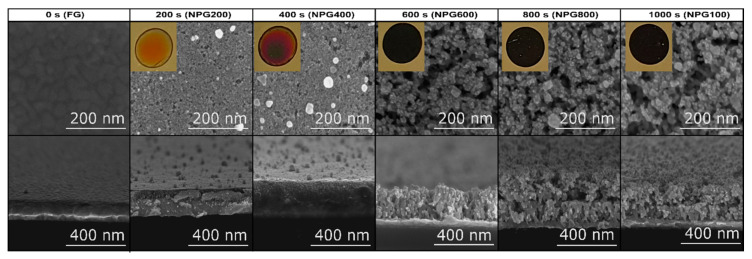
Top and cross-section scanning electron microscopy (SEM) micrographs of nanoporous gold (NPG) samples obtained after different etching times. Inset: photograph images of the experimental samples.

The NPG samples were prepared using as a starting substrate 4 inch silicon wafers (SIEGERT WAFER GmbH, Aachen, Germany) deposited with 20 nm chromium as an adherence layer and 300 nm gold by DC magnetron sputtering. Autolab PGCSTAT302n (Metrohm, Utrecht, The Netherlands) was used for NPG samples’ fabrication and for electrochemical analysis. A two-electrode configuration was employed, where the gold layer was used as a working electrode and round platinum grid with the diameter equal to the exposed area of the cell (0.5 cm^2^) as a counter electrode. Gold electrode was placed horizontally in the cell (Figure S1), while the platinum circular electrode was placed parallel to the surface at a height of approximately 2 mm away. The electrolyte contains equal volumes of 48% aqueous hydrofluoric acid (HF, analytical grade) and organic solvent dimethylformamide (DMF, 99.5% analytical grade). The volume of etching solution was 1 mL. The NPG samples were obtained using a 15 mA anodization constant current, and varying the process duration from 200 to 1000 s. The samples were labelled in the following manner: NPG200, NPG400, and so on, where the associated numbers are consistent with the process duration (Figure 1).

Scanning electron microscopy (SEM) micrographs were obtained using field emission gun scanning electron microscope (FEI Nova NanoSEM 630, Hillsboro, OR, USA). Absorption spectra were measured using integrating sphere in the wavelength range of 350–800 nm with a combined time resolved and steady state fluorescence spectrometer (Edinburgh Instruments, Livingston, UK). For the optical measurements, a black tape with a circular opening with the area of 0.5 cm^2^ where the porous layer is positioned was used in order to avoid the unwanted reflections from the remaining gold surface. The microstructure of the obtained samples, as well as the surface/pore morphology, were analyzed using a 9 kW Rigaku SmartLab diffractometer with rotating anode operated at 40 kV with 75 mA Small angle X-ray scattering (SAXS, Tokyo, Japan) was employed to reveal insights regarding the pore morphology, as well as to calculate the specific surface area. The electrochemical activity imaging was carried out under an ElProScan 3 Series Scanning Electrochemical Microscope (HEKA division of Harvard Bioscience Inc., Lambrecht (Pfalz), Germany) grounded in a Faraday cage and placed on an antivibration table, as well as with a piezoelectric nanopositioning system for fine approaches. The gold samples were soldered into a plastic Petri-dish (60 × 15 mm) with a back electrical connection suitable for kinetic studies that require substrate polarization. The SECM measurements were performed using a Pt counter electrode, Ag/AgCl reference electrode, and Pt ultramicroelectrode (UME) with a diameter of 10 μm and an RG of 5 (ratio between the insulator thickness (glass capillary that surrounds the electrode) and the radius of the electrode). The redox mediator solution employed in the experiments contained 3 mM FcMeOH (ferrocenemethanol) in 0.1 M KCl dissolved in ultrapure water. The reagents were purchased from Sigma-Aldrich (Darmstadt, Germany) and Alfa Aesar (Kandel, Germany), respectively. All chemicals were used without further purification.

## 3. Results and Discussions

### 3.1. Micro-Structural and Optical Investigations

The photographs presented as insets in Figure 1 show that the gold color changes with the increase in etching time, attesting to the shift of the metallic behavior towards longer wavelengths because of the decrease in the apparent plasma frequency caused by the shorter relaxation time of free carriers related to the scattering process in the material [30].

Thus, it can be clearly seen that, if the as deposited gold layer was initially reflective, after 1000 s of anodization, it becomes a perfect absorber in the visible spectrum. The SEM micrographs offer a thorough view of the gold anodization process evolution. Accordingly, the white islands present in the top-view images of the samples NPG200 and NPG400 are gold clusters formed on the sample surfaces by the removed atoms from the bottom of the pores. Advancing with the etching time, even though the color of the samples fabricated at 600, 800, and 1000 s only slightly increases in darkness, the analysis of the SEM micrographs using ImageJ software (v1.52a, NIH, USA) [31] reveals that the gold solid surface area decreases from 67% for NPG600 to 44% for NPG1000 (Figure S2). Moreover, the threshold level used in ImageJ for estimating the fractal dimension with the box counting method of NPG is depicted in Figure S2.

The gold anodization process recording correlated with the corresponding SEM images is also provided (Video S1). The in-depth evolution of the etching process is displayed in the transversal-view SEM images, where it can be observed that the thickness of the resulting porous layers increases from approximately 100 nm @ 200 s to 230 nm @ 1000 s, while the average pore surface area increased from almost 3 to 43% (Figure 1). As a result, the change of the color appearance with etching time is determined both by the increased porosity and the increase of the porous layer thickness.

**Figure 2:**
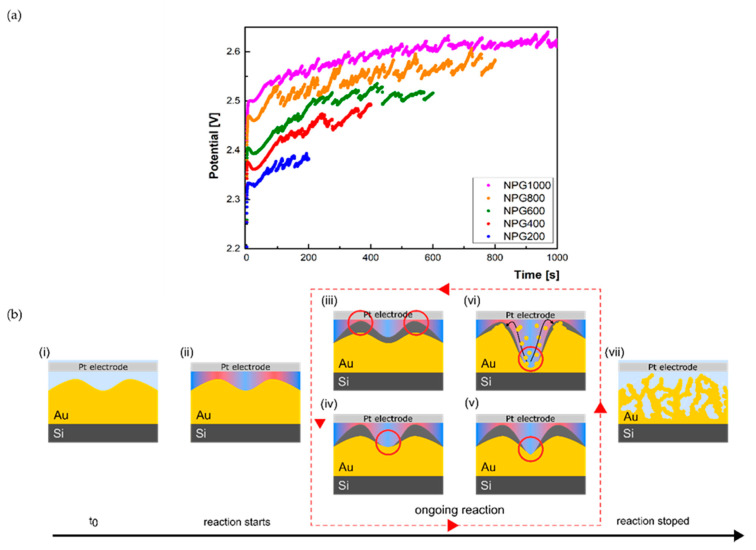
(**a**) The potential variation with etching time for a constant intensity of 15 mA (for clarity, the experimental points were translated vertically by 0.05 V) and (**b**) nanoporous gold formation reaction (i–vii).

Figure 2a shows typical voltage–time curves at a constant current density for the anodization of the Au substrate. Initially, voltage jumps (approximately 2.5 V) were observed corresponding to passive-to-active transition [32], followed by a temporary stagnation for a short period where an equilibration of the electrochemical system occurs. Thereafter, the voltage profile has a periodical saw-like shape profile that corresponds to a typical two processes’ competition. The voltage rise is associated with the formation of barrier oxide, whereas the voltage decay corresponds to the localized etching of the barrier layer that ultimately leads to the gold layer porosification [33]. Accordingly, the spikes in the voltage indicate practically when corrugations’ boundaries are “attacked” by HF, thus triggering the porosification process. As the process time increases, an increasing tendency is observed up to 400 s, where initialization of different porosification sites takes place, followed by a plateau characterized by in-depth pores’ evolutions, where an intense gas evolution from the sample surface was observed. The formation of pores is governed by the diffusion of reagents and is limited by the diffusion, as the agitation was not used during the etching process. A schematic illustration of this process mechanism is presented in Figure 2b. Thus, under anodic bias, an oxide layer is formed on the gold surface by consuming water to form Au(OH)*_x_* and H^+^ ions [3]—Figure 2b(i). Because of the surface corrugations, the electric field is stronger on top than at the bottom (at corrugations’ boundaries) (Figure 2b(ii)), increasing the reaction rate on top, thus leading to a thicker oxide layer on those areas (Figure 2b(iii)). The second reaction is represented by dissolving the oxide layer by the HF, this time with the reaction being faster at the bottom of corrugation because of the thinner oxide layer and the increased concentration of H^+^ transported from the top by diffusion and convection (Figure 2b(iv)). Whereas, during silicon porosification, a fluorosilane layer typically forms, AuF_4_ or AuF_3_ are formed in this case [34]. These compounds are instable and can decompose easily as Au(OH)_3_ in aqueous basic solution [35]. In order to increase the selective removal of Au(OH)*_x_* at the etching front, DMF was used as electrolyte to increase the stability of two compounds, AuF_4_ and AuF_3_ [36]. Therefore, at the etching front, a balance was established between oxidation and dissolution in that matter, that the resulting effect is the etching into depth of the gold surface, while the pore walls are protected by the reforming oxide (Figure 2b(v)). The difference between the two reactions’ speeds on top of the pore and at the bottom lead to the pore growing into depth (Figure 2b(vi)). The in-depth porosification practically represents a cyclic alternance of the (iii–vi) reaction steps. After the reaction stops, the remaining oxide layer is dissolved by the HF.

It is worth mentioning that we made a step further in validation of the proposed technology for the fabrication of the nanoporous gold, and we verified the robustness of the method for large-scale practical applications. Thus, using a 4 inch Si wafer size porosification system, we demonstrated the reproducibility of the proposed process, obtaining a highly uniform NPG layer on the roughly entire surface (Figure S3) that can then be subjected to standard photolithographic processes for more complex device fabrication.

**Figure 3:**
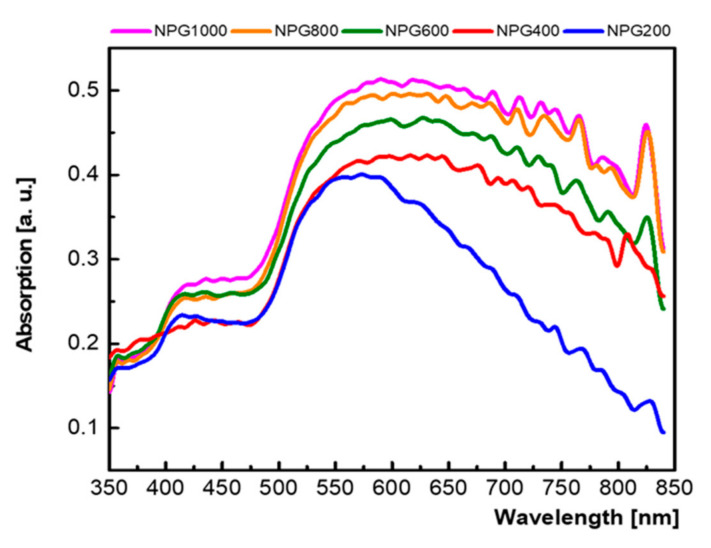
Absorption spectra of the NPG samples (the NPG spectra are normalized to the flat gold film absorption spectrum).

The optical properties of the nanoporous gold samples were investigated measuring the absorption spectra (Figure 3).

Two peaks are clearly defined, the first one in the 400–450 nm region that corresponds to the bulk gold plasmonic peak, and the second one at a wavelength beyond 500 nm assigned to localized surface plasmons, which depends on the morphology of the structure [37]. Taking into account that the NPG structures present a randomly distributed and different sizes of gold ligaments that support additional plasmonic modes, the broad absorption peak from longer wavelengths suffers a red shift with the increase of etching time. Moreover, the plasmonic resonances become stronger and the absorption peak increases. This effect could be attributed to the increase of both the porosity and the porous layer thickness (i.e., a lower porosity implies larger gold diameters, which, similar to the case of nanoparticles and nanorods, leads to a shift of the resonance wavelength).

Further, to complete the SEM surface investigations and to unravel the volumetric characteristics in terms of porosity and surface area of NPG, the SAXS analysis was employed.

Thus, for acquisition of the SAXS patterns, the incidence angle, *θ*, was varied from 0 to 2°. The scattering vector, *q*, defined using scattering angle (*θ*) and the incident X-ray wavelength (*λ* = 1.5406 Å), was calculated using Equation (1):

(1) $$q=\;\frac{4\pi sin\theta }{\lambda }$$

**Figure 4:**
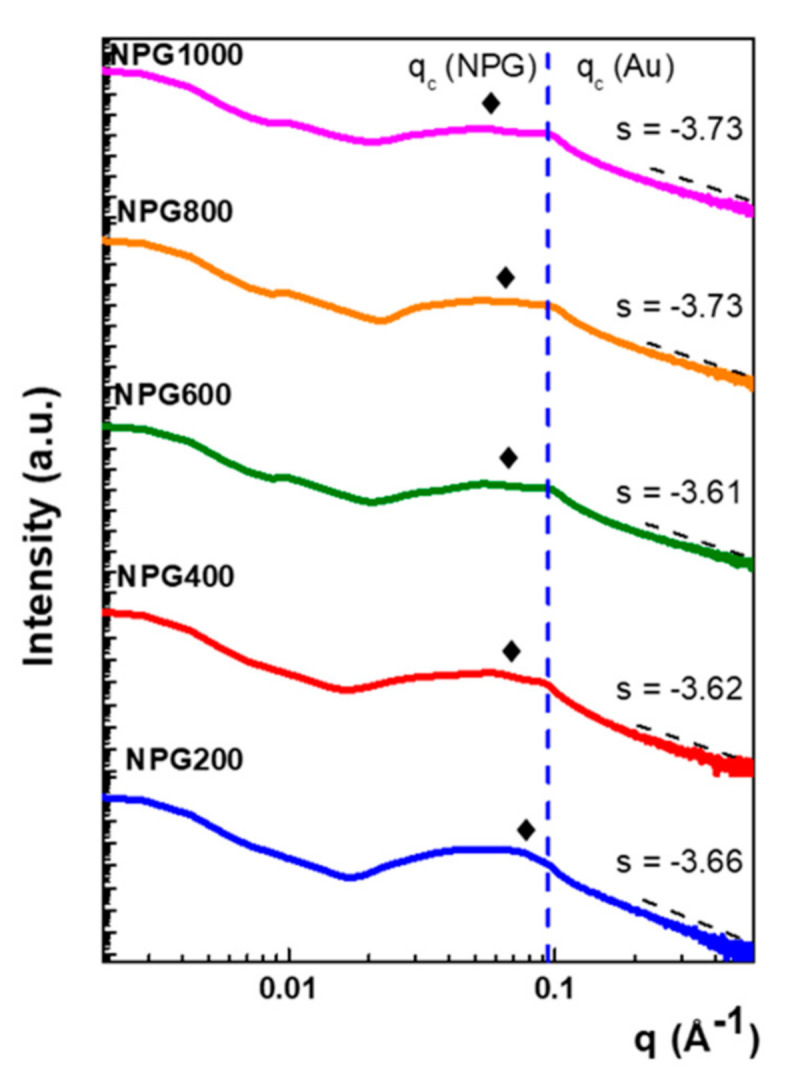
Small angle X-ray scattering (SAXS) patterns recorded for the investigated NPG samples. The blue dashed line indicates the critical angle of the gold, while diamonds stand for the corresponding ones for porous gold.

The resulting SAXS patterns of the intensity scattered by the investigated samples, expressed as a function of the scattering vector *q*, are presented in Figure 4.

Generally, two distinct domains arise in the SAXS patterns: on the one hand, the low-*q* scattering domain encodes the characteristics of crystalline domains examining the radius of gyration ($R_{G})$ and, on the other hand, the high-*q* domain obeys a power law and is related to the fractal dimension of the samples [38]. For instance, the slope (s) of high-q region ranges between −3.66 and −3.72, leading to a decrease of the fractal dimension, D, calculated as 6-s, from 2.34 to 2.27, which is related to an increase in the nanostructuration level.

In addition, it can also be observed that each SAXS pattern exhibits critical scattering vectors, where the intensity drops, which can be ascribed to the sample porosity. The position of the critical angle corresponding to bulk gold ($\theta_{c-Au}$) is preserved (illustrated with blue line), while the one related to the porous gold layer ($\theta_{c-NPG}$) suffers a shift towards smaller q values. The position of the critical scattering vector in the reciprocal space is: $q_{c}$ = 0.092 ${Å}^{-1}$ (blue dashed line in Figure 4 corresponds to bulk gold density, $\rho_{Au}$ = 19.3 g/cm^3)^. In the following, this critical angle will be denoted as *q_c-Au_*. At the same time, the values of the critical scattering vector that correspond to nanoporous gold *q_c-NPG_* successively decrease from 0.074 ${Å}^{-1}$ (NPG200), 0.065 ${Å}^{-1}$ (NPG400), 0.064 ${Å}^{-1}$ (NPG600), 0.062 ${Å}^{-1}$ (NPG800), and up to 0.055 ${Å}^{-1}$ for NPG1000. As the critical scattering vector for porous gold does not have a fixed value, the above values can be considered as average ones with a deviation of $\pm 0.002\;{Å}^{-1}$.

To relate the critical scattering vector to the sample density, the following calculations were performed considering that the complex index of refraction of matter for X-rays is close to unity [39]:

(2) n=1−δ−iβ

where δ is determined by the dispersion and β is proportional with the absorption coefficient having the following values [40]:

(3) $$\delta =\;\frac{\lambda^{2}}{2\pi }r_{e}\rho$$

(4) $$\beta =\;\frac{\lambda }{4\pi }\mu ,$$

where $r_{e}$ is the classical electron radius ($r_{e}$ = 2.8 × 10^−15^ m), ρ is the electron density, and μ is the linear absorption coefficient. In the assumption that the investigated materials has no absorption (β=0), the refractive index is expressed as follows:

(5) $$n_{layer2}=1-\delta$$

and the angle of the total reflection in the real space is given by the following [40,41]:

(6) $$\theta_{c}=\;\sqrt{2\delta }$$

Taking into account the relationship between the critical angle and the critical scattering vector (Equation (1)), as well as Equation (3), which relates δ coefficient by the sample density, we can express the sample density in the reciprocal space as follows:

(7) $$\rho =\frac{{\left(asin\left(\frac{\lambda q_{c}}{4\pi }\right)\right)}^{2}}{2}\frac{2\pi }{\lambda^{2}r_{e}}$$

Using the values obtained for the critical scattering vectors as well as its variation, the density of nanoporous gold was estimated, showing a decrease from 12.5 ± 0.2 g/cm^3^ to 6.29 ± 0.1 g/cm^3^. Finally, the NPG layer’s porosity was determined as follows:

(8) $$p=1-\frac{\rho_{NPG}}{\rho_{Au}}$$

Table 1 summarizes the values obtained for density and porosity calculated from SAXS patterns, as well as for average solid surface area ($\bar{p_{SEM}}$) and 2D fractal dimension ($D_{SEM}$) obtained from analysing the SEM micrographs.

As can be observed, both the porosity and average solid surface area values follow the same trend; the same tendency also arises for the fractal dimensions when determined from SEM and SAXS analyses. The differences between values arise from the probed area in each of the measurements: SEM only locally probes the surface sample morphology, whereas SAXS examines the entire volume of the sample, providing a more realistic view. The smaller values obtained for the fractal dimension by SEM practically confirm this observation, as the surface is generally more affected during the porosification process [42].

Further, to gain a quantitative framework of the specific surface area (*S_n_*), the Porod formalism was used, which is based on the following formulas:

(9) $$Q=\;\int_{0}^{\infty }I\left(q\right)q^{2}dq$$

(10) $$K_{p}=\;\underset{q\to \infty }{\lim}I\left(q\right)q^{4}\;\;$$

(11) $$S_{n}=10^{4}\frac{\pi p\left(1-p\right)}{\rho }\frac{Kp}{Q}$$

where *I(q)* is the scattering intensity; Q is the Porod invariant given by the Porod integral (9); $K_{p}$ is the Porod constant calculated with Formula (10) from the asymptotic behaviour of the tails in the high *q* region; and *p* and ρ are the sample porosity and density, respectively.

The *Iq* versus q and *ln(Iq^4^)* versus *q^2^* dependences, necessary for the calculation of the Porod integral and Porod constant, respectively, are presented in Figure S4. The results obtained for the Porod integral, Porod constant, and specific surface area are summarized in Table 2.

**Figure 5:**
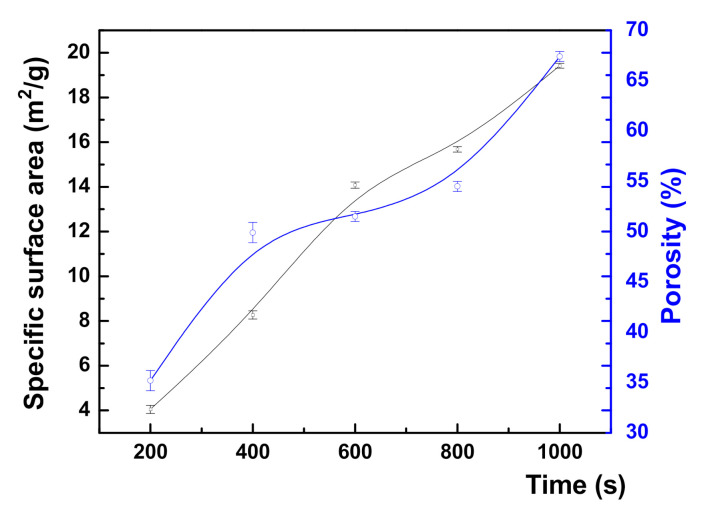
The dependence of the specific surface area (**black line**) and sample porosity (**blue line**) with increasing etching time. Error bars are used to show the standard deviation of the specific surface area and porosity estimated values.

They show distinct stages in the nanoporous gold layer formation at different etching times. In fact, an increase in the etching time led to significant modifications in the sample porosity and specific surface area (Figure 5). More precisely, with the increasing etching time, the NPG average porosity increases from 35.2 to 67.4%, which is associated with an increase of the specific surface area from 3.3 (for flat gold) to 3.67 for the thinnest NPG200 layer and further up to 17.96 m^2^/g for the thickest NPG1000 layer. It can also be observed that the specific surface area for NPG samples obtained at a higher etching time duration presents smaller deviations. This is reasonable taking into account the smaller variations of the porosity in the case of these samples, as shown in Table 1.

### 3.2. Electrochemical Characterization

Considering the huge potential of the nanoporous gold films towards electrochemical systems, cyclic voltammetry (CV) and electrochemical impedance spectroscopy (EIS) measurements were performed to evaluate the active surface area and interfacial properties. Thus, the electrochemically addressable surface area was firstly determined using the Au oxide reduction peak measured by CV and the corresponding charge associated with the reduction of gold oxide, by peak integration [43] (five cycles of CV were employed to certify that the response is stabilized).

**Figure 6:**
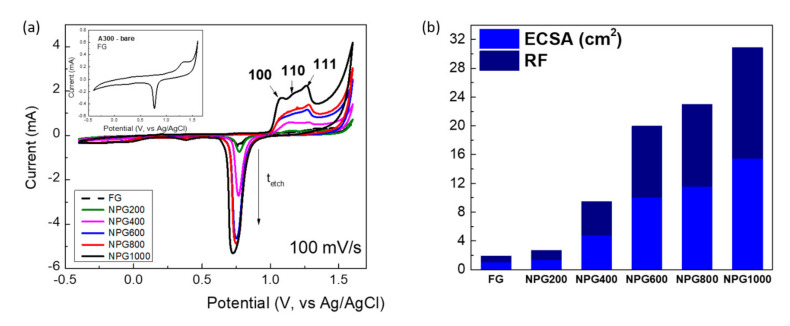
(**a**) Cyclic voltammograms (CVs) recorded with nanoporous Au electrodes in 0.5 M H_2_SO_4_ solution, at a scan rate of 100 mV/s (third cycle); (**b**) the electrochemical surface area (ECSA) and the roughness factor (RF) estimated from CVs.

The cyclic voltammograms were recorded in 0.5 M H_2_SO_4_ electrolyte, in the potential region from −0.4 to 1.6 V, at the scan rate of 100 mV/s (Figure 6a), and during potential cycling, anodic peaks between 1 and 1.3 V (vs. Ag/AgCl) and a well-defined cathodic peak at ~0.75 V were obtained because of the oxidation and reduction of the outermost layer of gold atoms. Whereas the flat gold surface exhibits a single oxidation peak (Figure 6a) due to the polycrystalline nature of the surface, three partially overlapped peaks arise after porosification determined by the oxide formation on the different crystallographic planes exposed, principally (100), (110), and (111) [44]. The corresponding electrical charge can be obtained by integrating the gold oxide reduction peak, which was converted into the electrochemical surface area (ECSA), assuming 390 μC/cm^2^ as the specific charge required for gold oxide reduction for polycrystalline gold [24]. Further, the roughness factor (RF) was calculated normalizing ECSA to the geometrical area (0.5 cm^2^). As can be observed in Figure 6b, the effective area of electrodes significantly increases with porosification duration, reaching 15.4 cm^2^ for the 1000 s period, revealing an approximately 16-fold enhancement of ECSA and RF compared with the standard flat Au electrode, significantly higher values than those reported for the nanoporous gold obtained via dealloying of Ag-Au alloy layers [45].

**Figure 7:**
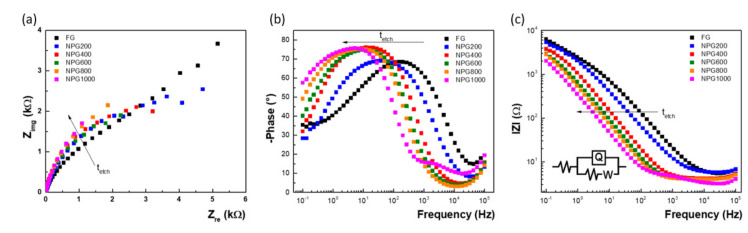
Electrochemical impedance spectroscopy results recorded with the flat Au and nanoporous Au electrodes in 0.5 M H_2_SO_4_ solution, at open circuit potential, from 100 kHz to 100 mHz using a 10 mV (rms) sine wave excitation: (**a**) Nyquist plots; (**b**) Bode plots—frequency dependence of the phase; and (**c**) Bode plots—frequency dependence of the imaginary impedance.

Next, the non-faradaic EIS was used to probe the porous films, measuring the double layer capacitance of the experimental test electrodes. As can be seen in Figure 7a, the Nyquist curves become steeper with the increase of the porosification duration, confirming the increase of capacitance. Concomitantly, the phase maximum successively increases, approaching 80°, and shifts towards lower frequencies—Figure 7b. The capacitive behavior is clearly shown in the intermediary frequency range, where the imaginary part of the impedance presents a linear variation as a function of frequency in log-log scale—Figure 7c. The slope of this linear part corresponds (with opposite sign) to the constant phase element (CPE) exponent that accounts for non-ideal porous electrode capacitance.

The impedance data were fitted considering an equivalent circuit for our system (inset image within Figure 7c containing the ohmic resistance of electrolyte (visible in the high frequency region) and the ionic charge transport resistance through the double layer and the double layer capacitance of the electrode, as well as the restricted diffusion of the electrolyte ions (W) [46] Data analysis was carried out using ZSimpwin software (v3.21, AMETEK, USA). The double layer capacitance values were obtained using the following formula:

(12) $$C_{dl}=\;Q^{1/n}\cdot R^{\left(1-n\right)/n}$$

where *Q* and *n* characterize the constant phase element and *R* is the associated resistance. 

**Figure 8:**
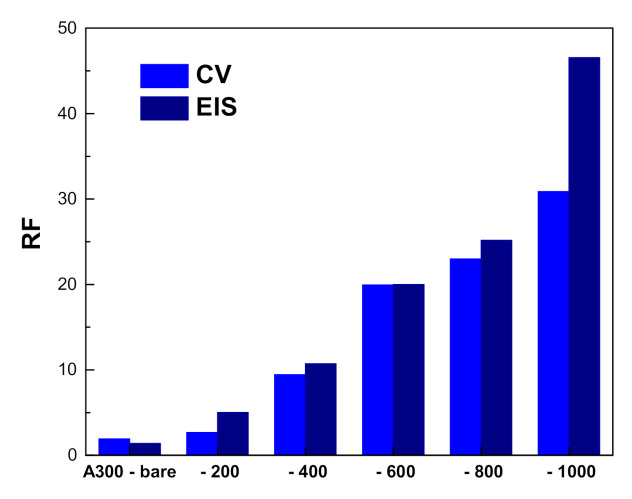
Roughness factor values estimated from CV and electrochemical impedance spectroscopy (EIS) measurements.

Relating the double layer capacitances determined by EIS measurements for the nanoporous gold to that corresponding to standard flat electrode, a roughness factor was also estimated [26], and the values obtained for the investigated electrodes using the two electrochemical techniques are shown in Figure 8. As can be observed, slightly higher values were obtained when the roughness factor was calculated using the EIS data, although they are more appropriate in comparison with previously reported results, where the presence of residual Ag fraction artificially increases the porous film double layer capacitance as its specific capacitance is larger than that of metallic Au [26,27]. As this issue is not present in our case, working with pure gold films, the EIS measurements provide an improved accuracy of values because the excitation signal reaches the bottom of the pores and senses the entire internal area of the porous electrode, not only the sites able to employ oxidation. It is, however, important to note that, strictly comparing our results with similar thickness samples obtained by dealloying processes based on chemical or electrochemical methods [19,47], a substantial simplification of the fabrication protocol was firstly demonstrated, as well as, furthermore, an increased porosity and consequently larger area of the electroactive surface with more than 30%.

**Figure 9:**
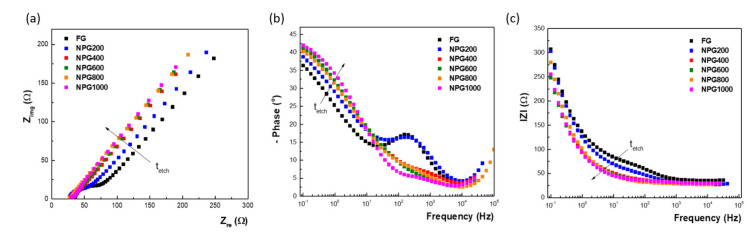
Electrochemical impedance spectroscopy results recorded with the bare Au and nanoporous Au electrodes in 2 mM Fe(CN)_6_^3^^−^/Fe(CN)_6_^4^^−^ phosphate buffer containing 0.1 M KCl, at the formal potential of the redox probe, from 100 kHz to 100 mHz, using a 10 mV (rms) sine wave excitation: (**a**) Nyquist plots; (**b**) Bode plots—frequency dependence of the phase; and (**c**) Bode plots—frequency dependence of the impedance module.

To complete the image of nanoporous gold electrodes, faradaic EIS measurements were also performed using ferri-ferrocyanide couple as redox probe aiming to characterize mainly the mass-transport to the electrodes associated with Faradaic processes that occur during charge injection with electrical stimulation. Thus, the EIS was carried out in biased voltage (formal redox potential of the ferri/ferrocyanide redox couple determined from CV measurements) and the recorded data are shown in Figure 9 in both Nyquist and Bode representations.

Remarkable differences can be observed when the porosification process is applied to obtain different thicknesses of the nanoporous gold layer. Thus, if the Nyquist plot initially consists of a distinct semicircle counting for the charge transfer resistance, which corresponds to the Bode phase peak in the intermediary frequency range, the radius of the semicircle starts to diminish after 200 s of porosification, moving the phase peak towards higher frequencies, and it practically disappears at higher process durations. In this case, we can talk about an extremely low electrode impedance, determined by the high surface area, and a correspondingly large charge injection capacity. Thus, the gold electrode nanostructuration led to enhanced surface area and conductivity.

The fitting of experimental data was done using a similar equivalent circuit, where the main parameter in the case of the faradaic EIS is the electron transfer resistance, which controls the electron transfer kinetics of the redox probe at the electrode interface. As a result, the heterogeneous electron transfer rate constant across the interface was obtained through the following relation [48]:

(13) $$k^{0}=\frac{RT}{n^{2}F^{2}AR_{ct}c}$$

where *R* is the gas constant, *T* is temperature in Kelvin, *n* is the number of moles of electrons transferred in the redox reaction (*n* = 1 for Fe(CN)_6_^3−/4−^), *F* is the Faraday constant, *A* is the geometrical area of the electrode (0.5 cm^2^), *R_ct_* is the charge transfer resistance determined from EIS, and *c* is the concentration of the redox couple.

**Figure 10:**
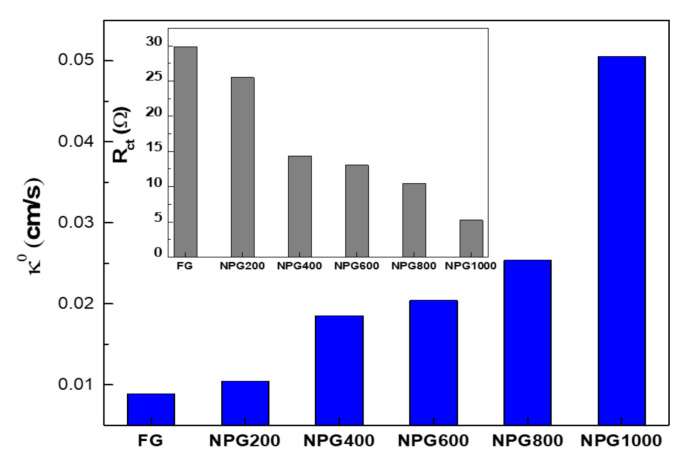
Heterogeneous electron transfer rate constant and electron transfer resistance (inset graph) determined from faradaic EIS measurements.

The obtained charge transfer resistance suggests the easing of charge transfer to and from the porous electrodes, also reflected by the continuous increase of *k^0^* values, which are larger in comparison with the previously reported results [48]. Thus, the heterogeneous electron transfer rate constant becomes 50 times higher for the thickest gold nanoporous layer, a similar order of increase as for the roughness factor (Figure 10).

Finally, the powerful spatial resolution of the scanning electrochemical microscopy technique [49] was employed as a cross-checking kinetic tool in the study of the nanoporous gold electrodes, practically achieving a correlation between the surface morphology investigated by SEM and the electrochemical activity. Experiments were performed using a 10 µm diameter Pt disk ultramicroelectrode in a 1 mM FcMeOH (0.1 M KCl electrolyte) solution.

**Figure 11:**
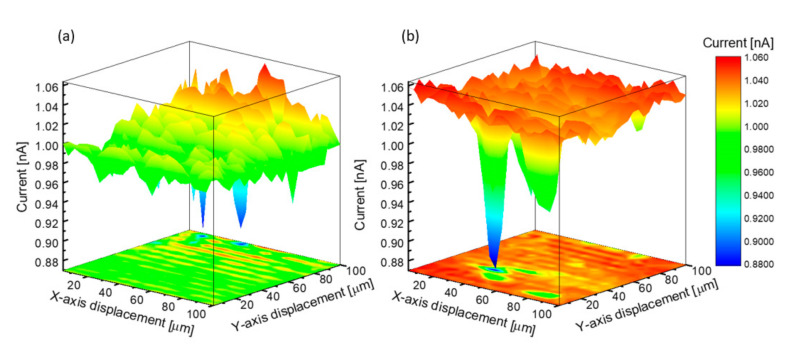
Scanning electrochemical microscopy (SECM) mapping images (100 × 100 µm) obtained in feedback mode when a tip potential of 0.35 V was applied: (**a**) Flat Gold (**b**) NPG800.

As shown in Figure 11, electrochemical details are unveiled in a spatially-resolved manner with the emphasis on the differences between the two substrates electrochemical response. Here, the 3D scans were performed over the nanoporous gold obtained after a 800 s anodization process in comparison with the initial flat substrate, showing the differences in electrochemical response in the presence of the redox mediator of the two unbiased substrates. It is worth mentioning here that the porous counterpart has higher current feedback zones because of the fact that many of the features formed during the process are suitable for the reduction of [FcMeOH]^+^. Moreover, the ligaments and the micropores enable a large surface area that drastically inhibits mass transfer of educts and products [50], which translates into a drop of feedback current as the tip scans over the surface. At the same time, the slow mass transfer on the overall reactivity of the ligaments and micropores makes the nanoporous gold substrate very attractive to many electrochemical applications. Therefore, the electrochemical investigations demonstrate the huge potential of nanoporous gold electrodes to be used for both non-faradaic electrochemical studies, where, generally, 1 kHz low impedance is required for neural electrophysiology to reduce the noise levels [51], and high sensitivity label-free affinity binding in faradaic-based biosensing, where high heterogeneous electron transfer rate constant is required [52].

## 4. Conclusions

We report a cost-effective, easily scalable, and controllable method to fabricate nanoporous gold films directly on silicon substrate for optical and/or electrochemical sensor applications that can be applied to other types of substrates, such as glass or flexible polymeric substrates. Moreover, adjusting the anodization current density, the nanoporous gold ligaments would be fine-tuned in order to achieve the desired properties. The careful calibration of the NPG fabrication process was complemented by a systematic characterization of the experimental samples in order to assess the evolution of the morpho-structural, optical, and electrochemical properties. The main drawbacks that other methods have, such as higher cost of fabrication, additional procedures of transferring onto a substrate, and residual material that could alter its properties, were overcome by this method of anodization at a lower voltage. The investigations show on the one hand the progressive increase of the sample porosity and specific surface area along with the etching time, and on the other hand the achievement of tunable optical and electrochemical performances that open new opportunities for the consistent development of novel devices based on NPG using a reliable wafer-scale process.

## Figures and Tables

**Table 1:** Morpho-structural properties determined using scanning electronic microscopy (SEM) and X-ray diffraction (XRD) investigations, where $\rho_{NPG}$ is the nanoporous gold density. p and D represent the porosity and fractal dimension of the sample calculated from SAXS patterns, respectively, whereas $\bar{p_{SEM}}$ and $D_{SEM}$ are the average solid surface area and 2D fractal dimension, respectively, obtained from SEM micrographs analysis. NPG, nanoporous gold.

Sample	$\rho {\;}_{NPG\;}(g/{\mathrm{cm}}^{3})$	p (%)	$\bar{p_{SEM\;\;}}\left(\%\right)$	D	$D_{SEM}$
NPG200	12.5 ± 0.2	35.2 ± 1	26 ± 3.15	2.34	1.92
NPG400	9.67 ± 0.2	49.9 ± 1	46 ± 1.08	2.38	1.86
NPG600	9.37 ± 0.1	51.5 ± 0.05	56 ± 1.32	2.39	1.76
NPG800	8.79 ± 0.1	54.5 ± 0.05	63 ± 1.6	2.27	1.71
NPG1000	6.29 ± 0.1	67.4 ± 0.05	66 ± 1.81	2.27	1.69

**Table 2:** Radius of gyration of ligaments (**R_g_**), size of the crystalline domains (**d_cryst._**), Porod integral (**Q**), Porod constant (**K_p_**), and specific surface area at different times (**S_n_**).

Sample	R_g_ (nm)	d_cryst._ (nm)	$Q\;({Å}^{-3})$	$Kp_{\;}({Å}^{-2})$	S_n_ (m^2^/g)
NPG200	20.3	57.4	122.75	0.87	3.67 ± 0.18
NPG400	22.1	62.5	59.83	0.61	9.44 ± 0.18
NPG600	18.0	50.9	68.37	1.15	15.77 ± 0.14
NPG800	16.9	47.8	63.27	1.12	17.70 ± 0.12
NPG1000	16.2	45.8	67.81	1.20	17.96 ± 0.11

## Supplementary Materials

The following are available online at https://www.mdpi.com/2079-4991/10/11/2321/s1, Figure S1: Experimental setup for nanoporous gold fabrication; schematic representation of the NPG etching system–cross-sectional view, Figure S2: The calculated average gold solid area (from SEM micrographs) for different etching time durations and the binarized images (insets), which were used for calculation of the fractal dimension in ImageJ by using box counting method, Figure S3: (a) 4 inch NPG on Si fabrication using AMMT system; (b) Photography of the resulted on wafer NPG film in comparison with the one obtained using microcell. Insets represents SEM top views of NPG in two distinct regions of the wafer, Figure S4: Iq vs. q and ln(Iq4) vs. q2 dependences for the: (a) Porod integral and (b) Porod constant, Video S1: Fabrication of NPG.
