# Supplementary material for: From Chip Size to Wafer-Scale Nanoporous Gold Reliable Fabrication Using Low Currents Electrochemical Etching
Source: Nanomaterials (Basel). 2020 Nov 23;10(11):2321. doi: 10.3390/nano10112321 (PMC7700230; doi:10.3390/nano10112321)
Supplement: Supplementary file 1 [file nanomaterials-10-02321-s001.pdf]

Supplementary Information

From chip size to wafer-scale nanoporous gold reliable fabrication using low currents electrochemical etching

Pericle Varasteanu [1,2,*], Cosmin Romanitan [1], Alexandru Bujor [1,3], Oana Tutunaru [1], Gabriel Craciun [1], Iuliana Mihalache [1], Antonio Radoi [1], and Mihaela Kusko [1,*]

[1] National Institute for Research and Development in Microtechnology (IMT-Bucharest), 126A Erou Iancu Nicolae Street, 077190 Voluntari, Romania; cosmin.romanitan@imt.ro (C.R.); alexandru.bujor@imt.ro (A.B.); oana.tutunaru@imt.ro (O.T.); gabriel.craciun@imt.ro (G.C.); iuliana.mihalache@imt.ro (I.M.); antonio.radoi@imt.ro (A.R.)

[2] Faculty of Physics, University of Bucharest, 405 Atomistilor Street, 077125 Magurele, Romania

[3] Faculty of Chemistry, University of Bucharest, University of Bucharest, 90-92 Panduri Street, 050663 Bucharest, Romania

* Correspondence: E-mail: pericle.varasteanu@imt.ro (P.V.); mihaela.kusko@imt.ro (M.K.); Tel.: +40-21-269.07.68

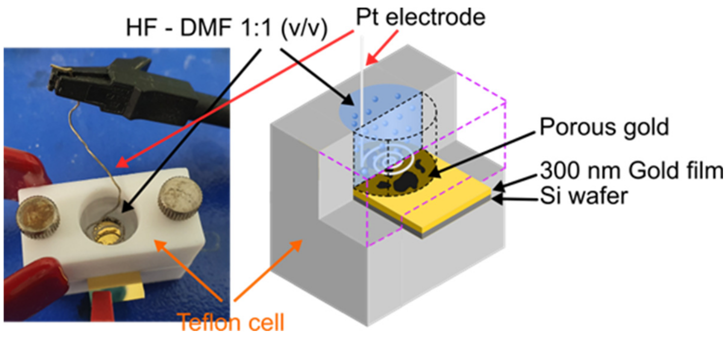

Figure S1. Experimental setup for nanoporous gold fabrication; schematic representation of the NPG etching system – cross-sectional view.

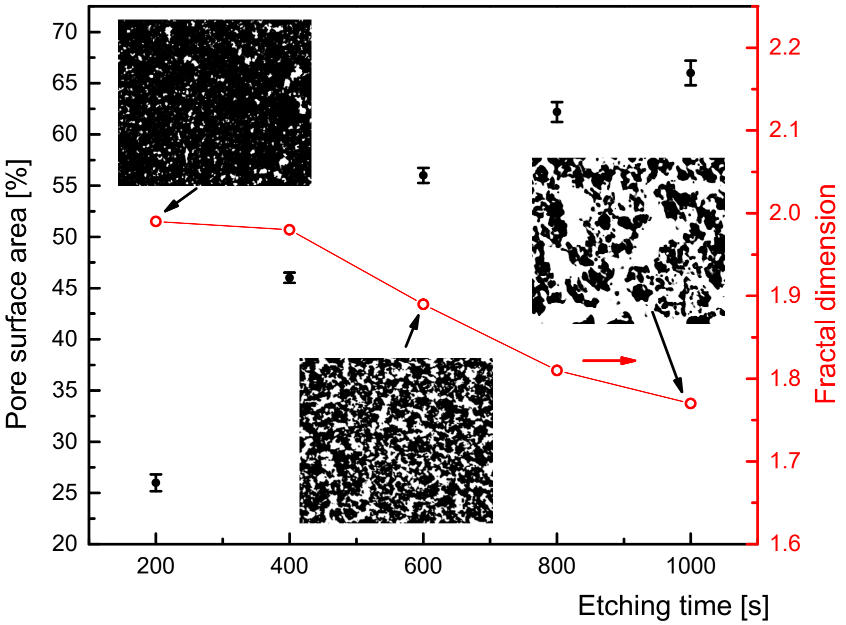

Figure S2. The calculated average gold solid area (from SEM micrographs) for different etching time durations and the binarized images (insets), which were used Figure 3. (**a**) 4 inch NPG on Si fabrication using AMMT system; (**b**) Photography of the resulted on wafer NPG film in comparison with the one obtained using microcell. Insets represents SEM top views of NPG in two distinct regions of the wafer.

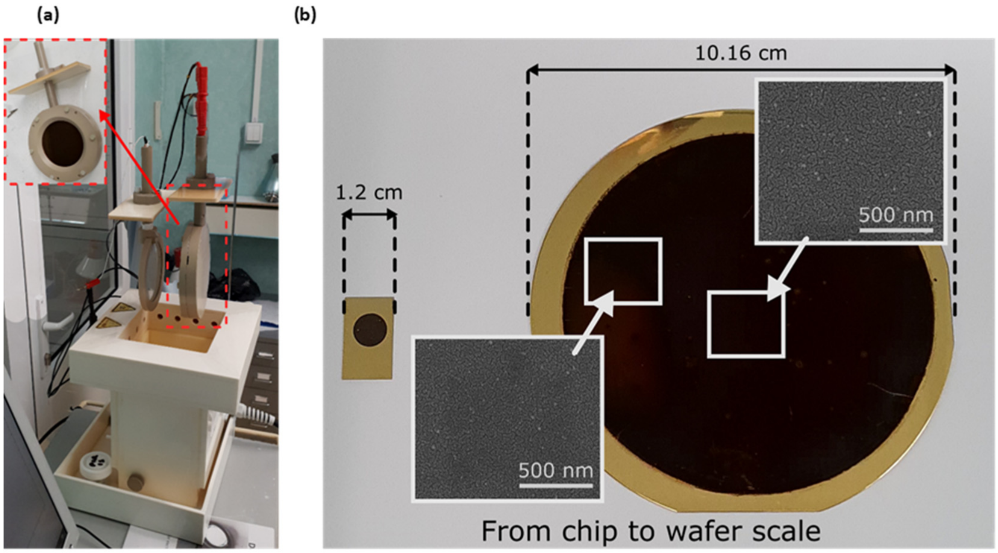

Figure S3. (**a**) 4 inch NPG on Si fabrication using AMMT system; (**b**) Photography of the resulted on wafer NPG film in comparison with the one obtained using microcell. Insets represents SEM top views of NPG in two distinct regions of the wafer.

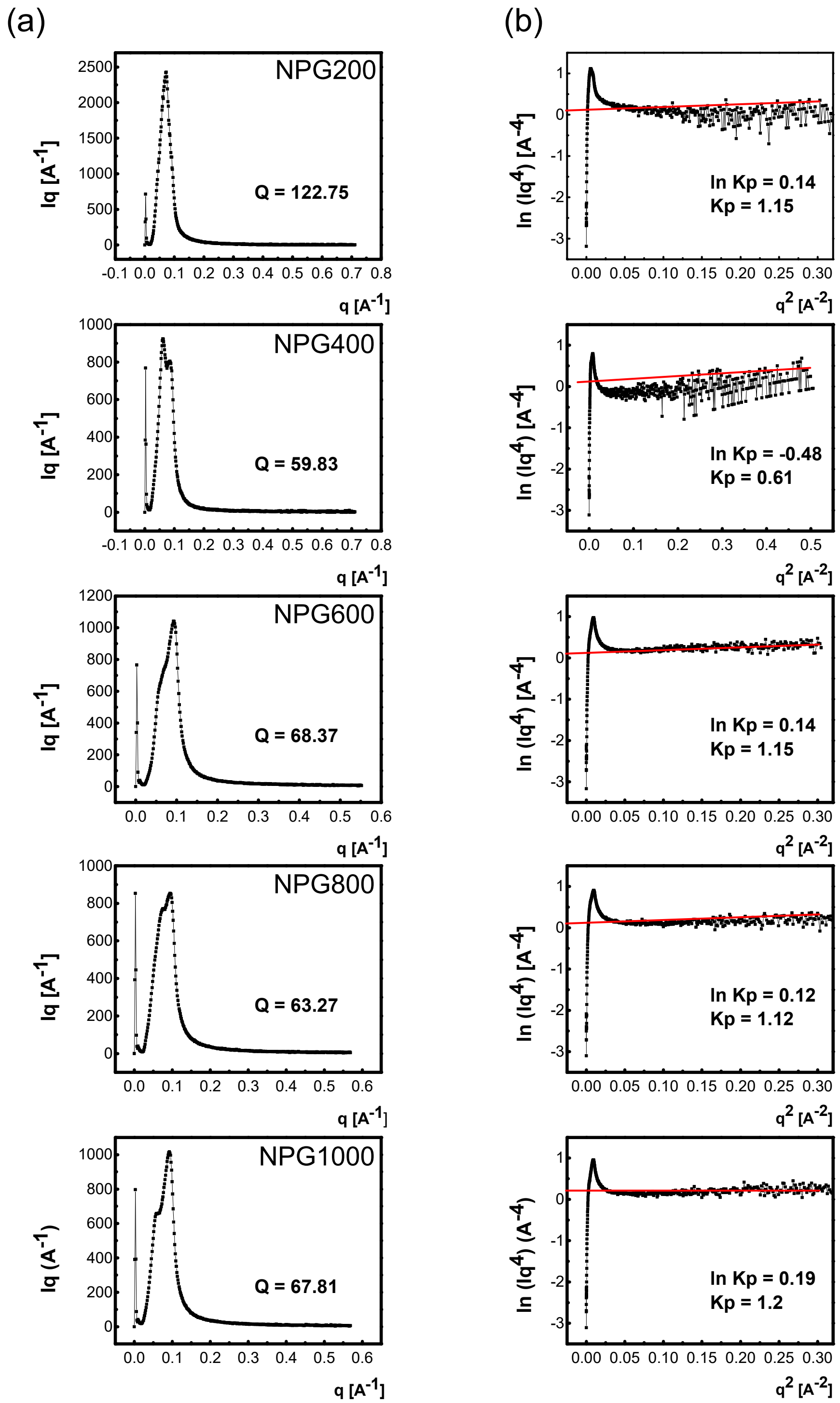

Figure S4: Iq *vs.* q and ln(Iq^4) *vs.* q^2 dependences for the: (a) Porod integral and (b) Porod constant.
